# Transmembrane Domain Length of Influenza a Virus M2 Does Not Determine Its Non-Lipid Raft Localization

**DOI:** 10.3390/v18010134

**Published:** 2026-01-21

**Authors:** Rashid Manzoor, Kosuke Okuya, Reiko Yoshida, Hiroko Miyamoto, Ayato Takada

**Affiliations:** 1 Faculty of Health Sciences, Higher Colleges of Technology, Sharjah P.O. Box 7946, United Arab Emirates; rmanzoor@hct.ac.ae; 2 Joint Faculty of Veterinary Medicine, Kagoshima University, Kagoshima 890-0065, Japan; kokuya@vet.kagoshima-u.ac.jp; 3 Division of Global Epidemiology, International Institute for Zoonosis Control, Hokkaido University, Sapporo 001-0020, Japan; zero-ysd-1102@nifty.com (R.Y.); hirom@czc.hokudai.ac.jp (H.M.); 4 International Collaboration Unit, International Institute for Zoonosis Control, Hokkaido University, Sapporo 001-0020, Japan; 5 One Health Research Center, Hokkaido University, Sapporo 060-0818, Japan; 6 Department of Disease Control, School of Veterinary Medicine, University of Zambia, Lusaka 10101, Zambia

**Keywords:** influenza virus, M2 protein, transmembrane domain, lipid raft

## Abstract

Influenza A virus expresses three envelope proteins, hemagglutinin (HA), neuraminidase (NA), and matrix protein 2 (M2). Of these, HA and NA associate with lipid rafts, whereas M2 remains in the peri-raft region. One reason for the lipid raft association of HA and NA is that they possess longer transmembrane domains (TMDs) (27 and 29 amino acids, respectively) than that of M2 (19 amino acids). Moreover, M2 localizes in the peri-raft region, despite the presence of some lipid raft-targeting features. Therefore, we introduced amino acid insertions into the N-terminal region of M2 to increase the TMD length to 22, 25, and 27 residues, and evaluated these M2-TMD mutants for their association with lipid rafts and impact on virus replication. Confocal microscopy, immunoprecipitation, and cell cytotoxicity assays showed that the cell surface expression and cytotoxic potential of M2-TMD mutants were comparable to those of wildtype M2. Based on the Triton X-100 solubility assay and colocalization analysis between lipid rafts and M2-TMD mutants, we found that the mutant proteins largely remained localized in non-raft domains. Importantly, an increase in M2-TMD length negatively influenced viral replication. These findings suggest that M2-TMD length is optimized for its proper function and does not determine its association with lipid raft domains.

## 1. Introduction

The genome of influenza A virus (IAV), a member of the *Orthomyxoviridae* family, comprises eight negative-sense RNA segments that encode 18 viral proteins, including three envelope proteins [1,2,3]; hemagglutinin (HA), neuraminidase (NA), and the matrix 2 (M2) protein [4]. IAVs are classified into subtypes based on various combinations of the two envelope glycoproteins, HA and NA. To date, 19 HA subtypes and 11 NA subtypes have been recognized [5,6,7,8]. The M2 protein is the smallest and one of the most extensively studied ion channel proteins. Four M2 monomers assemble to form a pH-regulated, proton-selective ion channel. Each monomer, consisting of 97 amino acids (aa), is divisible into an N-terminal ectodomain (ED, residues 1–24), a middle transmembrane domain (TMD, residues 25–43), and a C-terminal domain (CTD, residues 44–97) composed of an amphipathic helix and a cytoplasmic tail. The M2 protein plays multiple important roles in the IAV life cycle, both in ion channel activity-dependent and -independent manners from virus entry to virus release [1].

Cell membranes are laterally heterogeneous, comprising highly organized, cholesterol-rich, relatively rigid liquid-ordered (lipid raft) domains, and less organized, more fluid, liquid-disordered (non-raft) domains [9]. Lipid raft domains constitute detergent-resistant membrane (DRM) fractions rich in saturated phospholipids, glycosphingolipids, and cholesterol [10,11]. Lipid raft domains have been proposed to be involved in various important cellular processes, such as host and pathogen protein sorting, viral trafficking, and plasma membrane signaling [12,13,14,15]. Previous studies suggest that proteins use various signals for intracellular trafficking and sorting and possess specific cytosolic motifs for coat-mediated transport between organelles [16]. Particularly, hydrophobic TMD-lipid interactions have been shown to regulate plasma membrane trafficking [16]. It has also been shown that TMD length and specific sequences and/or motifs are critical determinants of protein-lipid raft association [17].

Budding of influenza viruses takes place at specialized regions of the plasma membrane known as budozones, formed by the coalescence of lipid raft domains [18,19]. The viral envelope proteins HA, NA, and M2 are synthesized in the endoplasmic reticulum (ER) and are transported to the plasma membrane via the trans-Golgi network [20]. Both NA and M2 use their TMDs as ER-targeting signals, whereas HA uses its N-terminal cleavable signal peptide for this purpose [21,22,23]. Interestingly, although IAV-infected cells express high levels of HA, NA, and M2 proteins on their surfaces, only HA and NA become major envelope proteins in virus particles. The reason for this preferential incorporation into virions is that both HA and NA proteins are targeted to the lipid raft domains, the sites for virus assembly and budding. Palmitoylation of the HA-TMD has been shown to be sufficient for recruiting HA to lipid rafts [24]. Similarly, the NA-TMD contains specific sequences and motifs that regulate the apical sorting and lipid raft association [22,25]. The M2 protein is also known to possess two raft association signals (i.e., palmitoylation of Cys50 and presence of the cholesterol recognition/interaction amino acid consensus motif) [26,27]. However, despite the presence of these lipid raft-targeting features, the M2 protein is excluded from lipid raft domains and remains localized in the peri-raft region. Thus, the relatively short TMD of M2 is thought to prevent its entry into lipid-raft domains [26]. Therefore, the present study aims to determine the relation between M2-TMD length and lipid-raft association, as well as its effect on virus replication.

## 2. Materials and Methods

### 2.1. Cells and Antibodies

Human embryonic kidney 293T (HEK293T) or human embryonic kidney 293 (HEK293) cells were maintained in Dulbecco’s modified Eagle’s medium (DMEM; Sigma-Aldrich, St. Louis, MO, USA) supplemented with 10% fetal calf serum. Madin-Darby canine kidney (MDCK) cells were maintained in minimum essential medium (MEM; Sigma-Aldrich, St. Louis, MO, USA) supplemented with 10% calf serum. An IAV strain, A/Puerto Rico/8/1934 (H1N1) (PR8), was obtained from our virus repository. Anti-M2 14C2 (Santa Cruz; Heidelberg, Germany), mouse anti-M1 monoclonal antibody (mAb) (APH 6-23-1-6) [28], mouse anti-HA mAb (APH 159-1-3), anti-β-actin (Abcam Limited, Cambridge, UK), and anti-α1 sodium potassium ATPase (Na^+^/K^+^-ATPase) (Abcam Limited, Cambridge, UK) were used as primary antibodies. Goat anti-mouse IgG (H + L) Alexa Fluor™ 488 (Invitrogen, ThermoFisher Scientific Inc; Waltham, MA, USA) and goat anti-mouse IgG (H + L) Alexa Fluor™ 594 (Invitrogen, ThermoFisher Scientific Inc; Waltham, MA, USA) antibodies were used as secondary antibodies as indicated elsewhere. HRP-conjugated goat anti-mouse IgG (H + L) antibody and goat anti-mouse IgM (H + L) (for anti-HA mAb) (Jackson ImmunoResearch, West Grove, PA, USA) were used as secondary antibodies in Western blot analysis.

### 2.2. Construction of Plasmids

The wildtype M2 (wtM2) gene was amplified from the cells infected with PR8 and cloned into the SacI and XhoI sites of the multiple cloning site of the eukaryotic expression vector pCAGGS/MCS (pCA) [29] to generate an M2-expressing plasmid (pCA-wtM2). The insertion mutations in the N-terminal region of M2-TMD were introduced using overlapping primers shown in Table S1. Primers PR8-M2_3aa ins, PR8-M2_6aa ins, and PR8-M2-42F-3 were used to generate PCR fragments having sequences for 3, 6, and 8 aa insertions into the M2-TMD, respectively. Remaining primers were used to generate the full-length M2 gene (Figure S1 and Table S1). Briefly, PCR was performed using KOD-Plus-Neo (TOYOBO Co., Ltd.; Osaka, Japan), and the amplified PCR products were digested with SacI and XhoI restriction enzymes, ligated into corresponding sites in pCA, and designated as pCA-M2-3aa, pCA-M2-6aa, and pCA-M2-8aa. The constructed plasmids were sequenced to confirm the presence of the desired mutations in M2-TMD and absence of unexpected mutations.

Recombinant M genes to generate infectious viruses were constructed using primers listed in Table S1, as shown in Figure S1. The amplified M genes containing M2-TMD insertion mutations were cloned into the pHH21 plasmid for viral genomic RNA expression [30]. We identified a unique restriction enzyme StuI site at nucleotide positions 738–743 in the PR8 M gene. The site was located in the sequence encoding the C-terminal region and the overlapping ED of M2, allowing us to introduce mutations in the M2-TMD without affecting M gene splicing. Two PCR fragments were amplified and ligated to produce full-length mutant M genes as outlined in Figure S1. In brief, a PCR fragment (positions 1–743) was obtained using a primer pair Bm-M-1 [31] and PR8-M-743R-StuI with pHH21-PR8-M serving as the template. The second PCR fragment (positions 738–1023) was amplified using a primer pair PR8-M-738F-StuI and PR8M-1003R with pCA-M2-3aa, pCA-M2-6aa, and pCA-M2-8aa serving as templates. In order to add an IAV RNA noncoding region and BsmB1 sites in the generated fragment, the amplified PCR products (positions 738–1003) were subjected to a second round of amplification using a primer pair PR8-M-738F-StuI and Bm-M-1027R [31]. The amplified PCR products were digested with StuI and BsmBI according to the manufacturer's instructions and ligated into the pHH21 vector using T4 ligase (Invitrogen, ThermoFisher Scientific Inc; Waltham, MA, USA) to generate plasmids pHH-PR8M2-3aa, pHH-PR8M2-6aa, and pHH-PR8M2-8aa.

### 2.3. Triton X-100 Solubilization Assay

MDCK cells were transfected with pCA-wtM2, pCA-M2-3aa, pCA-M2-6aa, or pCA-M2-8aa plasmids using Lipofectamine LTX (Invitrogen, ThermoFisher Scientific Inc; Waltham, MA, USA). The transfection medium was replaced with fresh growth medium three hours (h) later to minimize toxicity to the cells. Twenty-four h later, cells were washed with ice-cold PBS, and lysed with 1% Triton X-100 in NTE buffer (10 mM Tris HCl [pH 7.4]), 100 mM NaCl, 1 mM EDTA with protease inhibitors (Complete Mini cocktail) (Roch Diagnostics GmbH; Mannheim, Germany) on ice for 30 min (min) followed by centrifugation at 14,000× *g* for 30 min at 4 °C to separate soluble and insoluble fractions. The pellets were washed three times with PBS. Both the supernatants and pellets were adjusted to contain 1 × volume of radioimmunoprecipitation assay (RIPA) buffer and analyzed by Western blotting [32].

### 2.4. Surface Biotinylation and Immunoprecipitation

Cell surface biotinylation and immunoprecipitation (IP) assays were carried out with slight modifications to compare the cell surface expression of wtM2 and M2-TMD mutant proteins [33,34]. Briefly, MDCK cells were transfected with pCA-wtM2, pCA-M2-3aa, pCA-M2-6aa, pCA-M2-8aa, or pCA-PR8-HA plasmid as mentioned above. Twenty-four h later, the cells were washed three times with ice-cold PBS (pH 8.0). Cells were biotinylated in 0.5 mL PBS (pH 8.0) containing sulfo-NHS-SS-biotin (1.0 mg/mL) (Invitrogen, Thermo Scientific Inc; Waltham, MA, USA) for 30 min at 4 °C. The reaction was quenched by incubating cells with 50 mM glycine in PBS. Cell lysates were prepared in RIPA buffer, diluted at 1:2 with NTE buffer, and centrifuged at 14,000× *g* for 10 min at 4 °C. The supernatants were collected, and IP was carried out using streptavidin agarose beads (Millipore, Merck; Darmstadt, Germany). The streptavidin beads were washed three times with RIPA buffer. The beads were resuspended in Laemmli buffer and analyzed by Western blotting. Immunostaining of Na^+^/K^+^-ATPase was used as a loading control.

### 2.5. Cytotoxicity Assay

IAV M2-mediated cytotoxicity, an ion channel-dependent activity, was assessed by monitoring the loss of green fluorescent protein (GFP) signals as previously described [35,36]. Briefly, HEK293 cells were co-transfected with plasmids expressing enhanced GFP and wtM2 or M2-TMD mutants. Transfection with the GFP-expressing plasmid alone or with the PR8-NP-expressing plasmid was used as a control. Images were taken at 48 and 72 h post-transfection using a fluorescent microscope (Nikon Eclipse, TE2000-U, Nikon Corporation; Tokyo, Japan). EGFP-positive cells and mean fluorescence intensities were quantified using ImageJ software(version 1.54g) [37]. Data from three independent experiments are expressed as percentages relative to the mock control.

### 2.6. Immunofluorescence Assay

HEK293T cells grown in 8-chamber slides (Lab-Tek II chamber slide, Nunc, Thermo Fisher Scientific Inc; Waltham, MA, USA) were transfected with plasmids expressing M2 proteins. Twenty-four h later, cells were washed with PBS, fixed with 4% paraformaldehyde (PFA). Anti-M2 antibody (14C2) was used as a primary antibody, and goat anti-mouse IgG (H + L) Alexa Fluor 594 (Invitrogen, ThermoFisher Scientific Inc; Waltham, MA, USA) was used as a secondary antibody. M2 localization was also examined in permeabilized HEK293T cells using the same protocol, except that after fixation, cells were permeabilized with PBS containing 0.2% Triton X-100 for 5 min on ice. Nuclei were counterstained with DAPI. Colocalization of M2 with lipid rafts was examined using MDCK cells grown in 8-chamber slides (Lab-Tek II chamber slide). Cells were fixed with 4% PFA and stained without permeabilization. Cholera toxin subunit B (CTX-B) cross-linking was done using a Vybrant lipid raft labeling kit (Vybrant™ Alexa Fluor™ 488 Lipid Raft Labeling Kit, Invitrogen, ThermoFisher Scientific Inc; Waltham, MA, USA) according to the manufacturer’s protocol. After cross-linking, cells were fixed with 4% PFA and stained for CTX-B and M2 using anti-CTX-B and 14C2 antibodies, respectively. Goat anti-mouse IgG (H + L) Alexa Fluor™ 594 (Invitrogen, ThermoFisher Scientific Inc; Waltham, MA, USA) was used as a secondary antibody for M2 staining. The images were acquired as z-stacks with a 63x oil immersion objective on a Zeiss LSM780 confocal laser scanning microscope and analyzed using ZEN 2.6 Lite (Blue edition) software. The image stacks were used to construct three-dimensional (3D) images, which were then converted to the *xy*, *xz* and *yz* images.

### 2.7. Rescue of Viruses by Reverse Genetics

The twelve-plasmid reverse genetics system [30] was used to rescue PR8 viruses containing wtM2 and M2-TMD mutant genes. Briefly, HEK 293T cells were transfected with eight plasmids encoding viral RNAs together with the expression plasmids encoding viral nucleoprotein (NP) and viral polymerases PB2, PB1, and PA. Forty-eight hrs later, supernatants were collected and transferred to MDCK cells. MDCK cells were monitored for 72 hrs for the appearance of cytopathic effects (CPE). If no CPE or sign of virus growth in MDCK cells was observed, a second passage in MDCK cells was carried out. A portion of the cell supernatants was stored at −80 °C until use.

### 2.8. RNA Extraction, Reverse Transcription, and Real-Time PCR

RNA extraction, reverse transcription, and subsequent real-time PCR were carried out as described previously [38]. Briefly, HEK293T cells were transfected with the above-mentioned plasmids for virus rescue. Cell supernatants were centrifuged at 5000× *g* for 5 min at 4 °C, and then total RNA was extracted using Isogen (Nippon Gene Co., Ltd.; Tokyo, Japan) following the manufacturer’s protocol. The extracted RNA was treated with DNase I (Invitrogen, ThermoFisher Scientific Inc; Waltham, MA, USA), and the IAV NP gene in the extracted RNA was reverse-transcribed using SuperScript IV Reverse Transcriptase (Invitrogen, ThermoFisher Scientific Inc; Waltham, MA, USA) according to the manufacturer’s instructions with a sense-specific primer (NP-vRNA 5′–AGCAAAAGCAGGGTAGATAATCACTCAC–3′). One microliter of the mixture was used for real-time PCR using SYBR^®^ Premix Ex Taq II (Tli RNase H Plus) kit (Takara Bio Inc.; Kusatsu, Japan) and gene-specific primers NP-1186F (5′–ACCAATCAACAGAGGGCATC–3′) and NP-1333R (5′–TGATTTCGGTCCTCATGTCA–3′). The reaction conditions were as follows: 95 °C for 10 s (sec), followed by 40 cycles of 95 °C for 5 s and 60 °C for 30 s. The specificity of the primers was confirmed by dissociation (melting) curve analysis. One-step real-time PCR reactions were carried out on a QuantStudio™ 3 system (Applied Biosystems, Thermo Fisher Scientific Inc; Waltham, MA, USA), and data were analyzed using QuantStudio™ Design and Analysis Software (version 2.6.0).

### 2.9. Transmission Electron Microscopy

Cell supernatants from HEK293T cells transfected with plasmids for virus rescue were collected for electron microscopy. Samples were prepared as described previously with slight modifications [39]. The collected supernatants were centrifuged at 5000× *g* for 10 min to remove cellular debris, followed by centrifugation at 30,000× *g* for 90 min. The resulting pellets were resuspended in PBS containing 0.1% sodium azide. The pellets were gently resuspended, fixed with 0.25% glutaraldehyde, adsorbed onto collodion-carbon-coated copper grids, and negatively stained with 2% phosphotungstic acid solution (pH 5.8). Samples were examined with a transmission electron microscope (Hitachi, H-7800, Hitach High-Tech Corporation; Tokyo, Japan) operated at 80 kV.

## 3. Results

### 3.1. Design of M2-TMD Mutants and Their Properties

**Figure 1:**
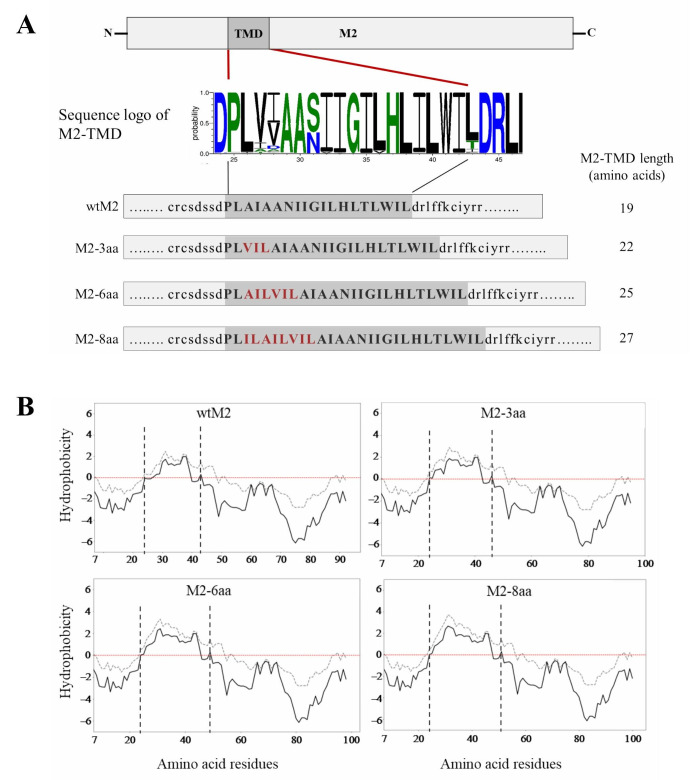
(**A**) Schematic diagram of M2 and its TMD. A total of 6413 M2 amino acid sequences of H1–H15 HA subtype influenza A viruses were retrieved from GenBank using the “collapse identical sequences” function. Multiple sequence alignment was performed with UGENE software (version 1.27.0), and the resulting alignment was used to create an M2-TMD amino acid sequence logo with the WebLogo 3 online tool [44,45]. Amino acid residues inserted into the N-terminal region of M2-TMD are shown in red. (**B**) Hydrophobicity analysis of wtM2 and TMD-mutant proteins. The analysis was carried out for the sequences from position 7 to the C-terminus according to Kyte & Doolittle [42] (grey dashed line) and Engelman et al. [43] (black solid line) with scales over a window of seven residues. Paired vertical dashed lines indicate the position of the TMDs in wtM2 and M2 mutants. On the plot, positive and negative peaks, above and below the red dotted line, indicate the likelihood that the corresponding polypeptide fragments are hydrophobic and hydrophilic, respectively.

HA and NA, which localize in lipid raft domains, possess 27- and 29-amino-acid-long TMDs, respectively. By contrast, M2 localizes in non-lipid raft domains, and its TMD is 19-amino-acid-long. It has been reported earlier that TMD lengths of membrane proteins act as cytosolic and/or membrane sorting factors [40], suggesting that the relatively shorter M2-TMD might lead to peri-raft localization. Therefore, to investigate this possibility, we introduced insertions of 3, 6, and 8 amino acids in the N-terminal region of the M2-TMD to increase their lengths to 22, 25, and 27 amino acids (Figure 1A). The N-terminal region of the M2-TMD is highly conserved among IAVs (Figure 1A); therefore, we introduced the amino acid insertions into the N-terminal end of the ion channel. Another reason for introducing insertion mutations at the N-terminal end was that the C-terminal end contains the functional core of the ion channel formed by residues His37–Trp41 [1,41]. The amino acids for the insertion were selected based on their frequency in the N-terminal domain as shown in Figure 1A. We further examined the effects of the amino acid insertions on the predicted hydrophobicity of TMD using Kyte and Doolittle [42] and Engelman [43] hydrophobicity scales (Figure 1B). These analyses showed that amino acid insertions into the M2-TMD did not alter its overall hydrophobicity, and the amino acid insertions likely extended the lengths of the hydrophobic TMDs in the mutant M2 proteins.

### 3.2. Membrane Localization of M2 and Its TMD Mutants

**Figure 2:**
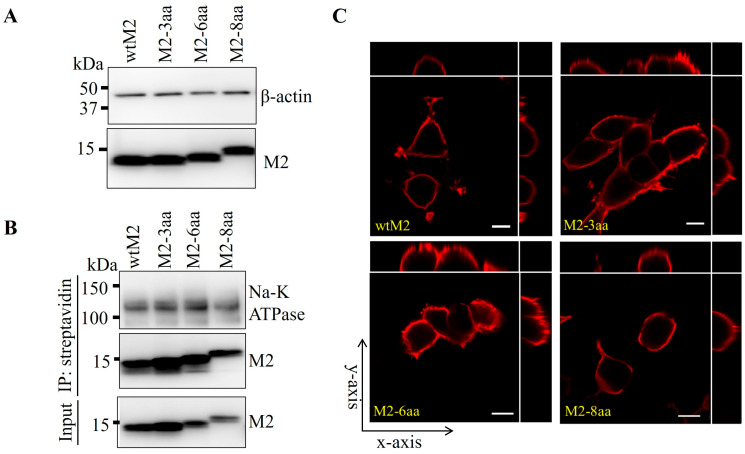
Expression of M2 and its TMD mutants on cell surfaces. (**A**) Western blot analysis of wtM2 and M2-TMD mutants expressed in HEK293T cells transfected with plasmids pCA-wtM2, -M2-3aa, M2-6aa, or M2-8aa. β-actin was used as a loading control. (**B**) MDCK cells were transfected with pCA-wtM2, -M2-3aa, M2-6aa, or M2-8aa expression plasmids. Twenty-four h later, cell surface proteins were biotinylated, quenched, and cell lysates were prepared. Biotinylated proteins were immunoprecipitated using streptavidin agarose beads and analyzed by SDS-PAGE followed by western blotting. Na^+^/K^+^-ATPase was used as a membrane marker protein. (**C**) Immunofluorescent staining of HEK293T cells transfected with expression plasmids described above. At 24 h post-transfection, cells were fixed, blocked, and stained with an anti-M2 monoclonal antibody and goat anti-mouse IgG (H + L) Alexa Fluor™ 594 secondary antibodies. Images were acquired as z-stacks and converted to the *xy*, *xz* and *yz* planes. Scale bars indicate 10 µm.

HEK293T cells were transfected with expression plasmids encoding wtM2 and M2-TMD mutants, and their intracellular and cell surface expression levels were compared. Western blot analysis of cell lysates showed that expression levels of wtM2 and M2-TMD mutants were similar (Figure 2A). Next, we assessed the cell surface expression of these proteins by IP and confocal microscopy. Cell surface proteins of the transfected cells were biotinylated and precipitated using streptavidin beads and analyzed by Western blotting. It was found that wtM2 and TMD mutant proteins precipitated to similar levels, suggesting that M2-TMD mutants were targeted to the cell surface as efficiently as wtM2 (Figure 2B). Confocal microscopy of the transfected HEK293T cells also revealed that all tested M2 proteins were present on cell surfaces (Figure 2C and Figure S2). In conclusion, these results showed that increased TMD lengths did not affect cell membrane localization of M2 proteins.

### 3.3. Ion Channel Activity of M2 and Its TMD Mutants

**Figure 3:**
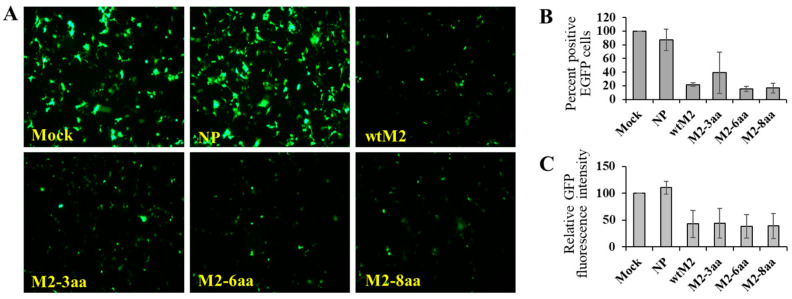
Viability of cells expressing M2 and its TMD mutants. (**A**) HEK293 cells were co-transfected with pCA-EGFP together with empty vector (Mock), pCA-NP, pCA-wtM2, pCA-M2-3aa, pCA-M2-6aa, or pCA-M2-8aa. Images were captured at 48 h post-transfection (fluorescence microscope, 10× objective). (**B**) Quantification of EGFP expressing cells and (**C**) relative fluorescence intensities compared to mock as an indicator of ion channel activity.

It has been shown that treatment of cells coexpressing M2 and GFP with a low-pH buffer activates the ion channel activity of M2, resulting in cell death and loss of GFP from the cytoplasm [36]. We employed this assay to ascertain whether the longer M2-TMDs affect its ion channel activity. HEK293 cells transfected with the GFP-expressing plasmid alone or together with the NP-expressing plasmid showed no significant change in GFP signals at 72 h post-transfection. By contrast, a loss of fluorescence was evident in cells expressing wtM2 and M2-TMD mutants (Figure 3), consistent with previous studies showing that this cytotoxicity is dependent on the M2 ion channel activity [35,36]. Thus, these findings suggested that changing the M2-TMD length did not affect the ion channel function of M2.

### 3.4. Lipid Raft Association of M2 and Its TMD Mutants

**Figure 4:**
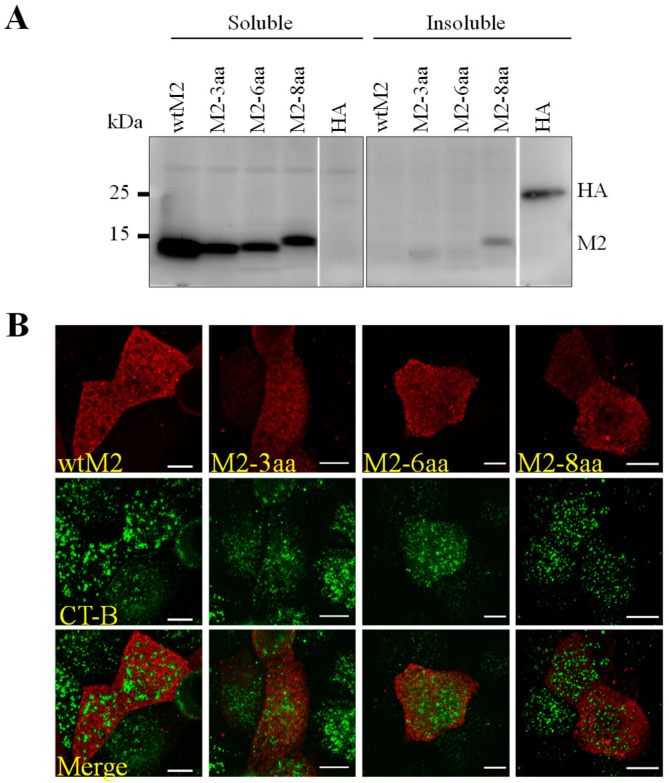
Independence of M2 localization from TMD length. (**A**) MDCK cells were transfected with plasmids expressing wtM2, M2-3aa, M2-6aa, M2-8aa, or HA proteins. Cells were lysed with 1% Triton X-100, and the lysates were separated into soluble and insoluble fractions by centrifugation, followed by western blot analysis. (**B**) MDCK cells were transfected with plasmids pCA-wtM2, pCA-M2-3aa, pCA-M2-6aa, or pCA-M2-8aa. Cell surface proteins were cross-linked with CTX-B and anti-CTX-B, then fixed, and stained for CTX-B and M2 proteins. Scale bars indicate 10 µm.

We then tested whether the amino acid insertions into the TMD affect the association of M2 with lipid rafts using a Triton X-100 solubility assay (Figure 4A). This assay has been extensively used to study the association of glycosylphosphatidylinositol (GPI)-anchored proteins such as CD59 [46] and IAV HA and NA with lipid rafts [47,48]. We found that both wtM2 and M2-TMD mutants mainly remained in the lipid-soluble fraction, whereas HA mainly remained in the lipid-insoluble fraction. To further confirm the non-lipid raft association of M2 and its TMD mutants, MDCK cells transiently expressing wtM2 or M2-TMD mutants were analyzed for colocalization with CTX-B as a lipid-raft marker (Figure 4B). Similar to wtM2, which did not colocalize with CTX-B, no significant effect was found on the localization of M2-TMD mutants. These results suggested that wtM2 and M2-TMD mutants localized to the non-lipid raft regions.

### 3.5. Effects of Altered M2-TMD Length on Virus Rescue and Replication

**Figure 5:**
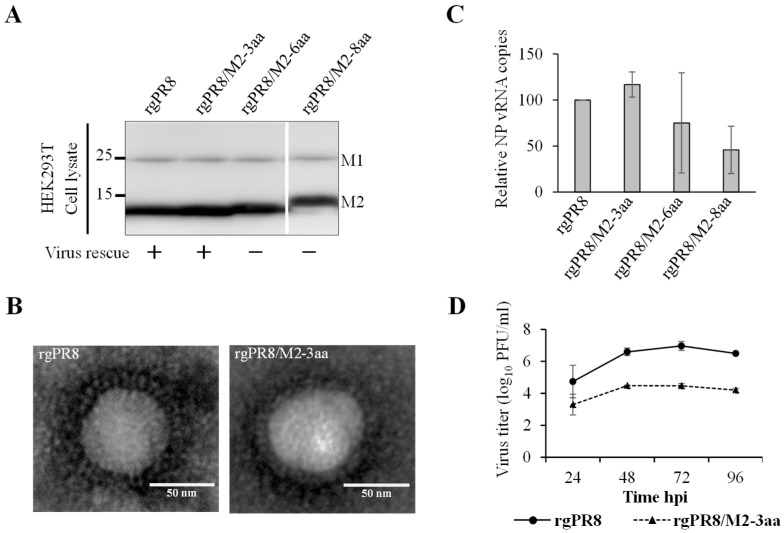
Properties of rescued PR8 viruses. (**A**) HEK293T cells were transfected with plasmids for virus rescue as described in the Materials and Methods section. The transfected cells were tested for the expression of viral M1 and M2 proteins using western blotting. (**B**) Virus particles pelleted from supernatants of transfected HEK293T cells were negatively stained and examined by electron microscopy. (**C**) Viral RNA was extracted from transfected HEK293T cells and treated with DNase, and the NP vRNA levels were measured using real-time PCR. The NP vRNA levels were normalized to rgPR8 NP levels. The results were based on two independent experiments performed in triplicate. The error bars represent the standard error. (**D**) MDCK cells were infected with rg-wtPR8 and rg-PR8/M2-3aa viruses at a multiplicity of infection of 0.01. Cell supernatants were collected at the indicated time points and virus titers were determined by plaque assays.

Finally, we examined the impact of the extended M2-TMD on viral replication in vitro. Attempts were made to rescue recombinant viruses carrying wtM2 or M2-TMD mutants by using a 12-plasmid reverse genetics system [30]. However, we were able to rescue only the parental PR8 (rgPR8) and PR8 carrying the M2-3aa mutant gene (PR8/M2-3aa). In all instances, the virus rescue attempts were repeated three times under carefully-controlled conditions to ensure consistency. To confirm that the failure to produce other mutant viruses was not due to aberrant splicing of the M gene transcript, we examined the expression of M1 (the primary protein expressed from the non-spliced M gene mRNA) and M2 in HEK293T cells transfected with all plasmids for virus rescue (Figure 5A). Western blot analysis clearly showed that M gene mRNA splicing occurred, as indicated by similar expression levels of M1 and M2 proteins among the transfected cells (Figure 5A). Then, the supernatants were ultracentrifuged and subjected to electron microscopy. We observed virus particles only in the supernatants of cells from which rgPR8 and rgPR8/M2-3aa viruses were rescued (Figure 5B). The levels of NP viral RNA were mostly comparable between cells transfected to rescue rgPR8 and rgPR8/M2-3aa viruses, whereas the vRNA levels of rgPR8/M2-6aa and rgPR8/M2-8aa were slightly lower (Figure 5C). Finally, we compared the growth kinetics between rgPR8 and rgPR8/M2-3aa (Figure 5D). We found that the titers of rgPR8/M2-3aa were significantly lower than those of rgPR8 at all time points. Our virus growth kinetics and virus rescue results suggested that an increase in M2-TMD length might impair virus growth by reducing virus release from infected cells.

## 4. Discussion

Proteins possess many types of cytoplasmic organelle-specific signals for adaptor/coat-mediated sorting such as lipid interactions through hydrophobic TMDs. Previous studies have shown that a strong correlation exists between TMD lengths and organelle specificity: Proteins with a short TMD tend to be targeted to the Golgi complex or endoplasmic reticulum, while proteins with a long TMD tend to be transported to plasma membranes [40,49,50]. Evidence suggests that the plasma membrane is laterally heterogenous comprising highly ordered lipid raft domains and less organized, more fluid, disordered, non-lipid raft domains. The ordered cholesterol-rich regions have been shown to be 0.6–1.5 nm thicker than the disordered domains [17,51]. Previous studies have shown that proteins with longer TMDs destined for the plasma membrane tend to show affinity for these ordered, lipid raft domains [40,52,53]. Therefore, in the present study, we hypothesized that a change in the M2-TMD length might affect its lateral sorting in the plasma membrane.

Generally, two approaches have been used to study the interaction between the plasma membrane and TMDs: Altering the membrane thickness or changing the TMD lengths of proteins. The thickness of the lipid bilayer can be altered by using artificial bilayer membranes made up of amphiphilic phospholipids with the ability to control the membrane thickness by changing the lipid acyl chain length. Many platforms are available in which lipid bilayers are created, including bilayer membranes or unilamellar lipid vesicles (ULVs) [54]. However, altering the lipid bilayer thickness affects not only the membrane thickness but also the lipid composition of both domains (lipid-raft and non-lipid raft domains), which could potentially influence the results. In fact, most of the studies conducted to understand the characteristics of the M2-TMD in plasma membranes used ULVs, giant unilamellar vesicles, synthetic lipid bilayers, or M2-TMD-truncated mutants [55,56,57,58,59]. In contrast to these conventional approaches, varying the TMD lengths of proteins has offered more reliable results than changing the thickness of artificial bilayer membranes [60]. Therefore, we also opted for this approach to increase the length of the M2-TMD by adding hydrophobic residues to its N-terminal region.

TMD lengths of membrane proteins have been shown to be important for regulating their membrane localization. Shortening the TMD length of HA from 27 to 17 amino acids resulted in the translocation of 80% of the mutant protein to the Golgi and ER [61]. Moreover, a reduction in the TMD length of syntaxin-3 from 25 to 17 amino acids resulted in total retention of this protein in the cis-Golgi compartment, preventing its transport to the plasma membrane [62]. Another study demonstrated that lengthening the TMD of perfringolysin-O increased its affinity for lipid raft domains, whereas shortening it weakened the interaction compared with the wildtype [60]. However, our study showed that increasing the M2-TMD length did not affect its association with non-lipid raft domains in the plasma membrane. The most plausible explanation for this phenomenon could be the presence of specific motifs in the TMD that regulate membrane sorting of M2. In fact, it has been shown that conserved amino acid motifs in TMDs of some proteins govern membrane sorting as well as homo- and heterodimerization of multimeric proteins [63]. For example, a conserved Gln residue in the TMD of N-acetylglucosaminyltransferase I (GnTI) determines its cis/medial Golgi localization [64]. Mutations in the HA-TMD at positions 507, 511, 517, 520, and 521 severely affect the association of HA with lipid rafts [21]. Peripheral myelin protein 22 (PMP22), which exhibits a pronounced preference for liquid ordered domains, shifts its preference to liquid-disordered domains upon mutations at positions 69 and 118 in its TMD [65]. These studies clearly demonstrate that, in addition to TMD lengths, specific amino acids in TMDs also regulate membrane sorting of proteins. In case of the M2 protein, studies have shown that despite the presence of the cholesterol-recognition amino acid consensus motif in the amphipathic helix region, the M2 protein preferentially partitions in non-lipid raft domains [26,66]. Therefore, there is a possibility that specific amino acids in the TMD might regulate the sorting of M2 into non-lipid raft domains. However, further studies are needed to clarify this mechanism.

The M2 protein plays an important role in the process of virus budding and release [59]. In our study, it was found that an increase in the M2-TMD length had a negative impact on the viral life cycle, as indicated by the significant reduction in the growth of rgPR8/M2-3aa compared to the rgPR8 virus and failure to rescue rgPR8/M2-6aa and rgPR8/M2-8aa viruses. Due to the inherent propensity of the M2-TMD to remain in non-lipid raft domains, increased TMD lengths may simply result in a hydrophobic mismatch. Different consequences of hydrophobic mismatch have been proposed for the arrangement/orientation of TMDs of proteins in the membrane environment [53]: (i) stretching of lipid acyl chains in the direct vicinity of TMDs to accommodate a long TMD [67], (ii) self-association of TMDs [68], (iii) deformation of the TMD backbone to decrease the effective length of peptides, (iv) kinking or flexing in the TMD backbone [69], or (v) possible binding of TMDs to membrane interfaces (i.e., a non-transmembrane state) [70]. Based on these, it is plausible that alteration of the M2-TMD may lead to conformational and/or orientational changes that affect the abilities to assemble into homotetramers and/or to facilitate virus egress. In fact, it has been shown that the M2-TMD undergoes coupled conformational changes in pore-facing amino acid residues due to changes in bilayer thickness, drug binding, or pH. These structural changes could be attributed to the formation of a well-defined helical kink at Gly34 observed in thick lipid bilayers and the amantadine-bound TMD [71,72]. Previous studies have demonstrated that the M2 cytoplasmic tail plays a crucial role in infectious virus production by facilitating the efficient packaging of viral ribonucleoproteins (vRNPs) into influenza virus particles [73,74]. The M2 cytoplasmic tail has also been shown to directly interact with the M1 protein, thereby facilitating the recruitment of vRNPs during virus assembly [75,76]. Therefore, it is also possible that disruption of the M2 structure, even within the transmembrane domain, may allosterically affect the conformation of the cytoplasmic tail and compromise its ability to efficiently interact with vRNPs, either directly or via the M1 protein, ultimately affecting the production of infectious virus particles.

## 5. Conclusions

The present study demonstrated that increasing the M2-TMD length did not alter the localization of M2 to non-lipid raft domains, while such structural modifications negatively affected viral replication and release. These findings suggest that membrane sorting of M2 is not simply governed by TMD length but may depend on specific amino acid residues and/or motifs that modulate its interaction with the membrane environment. Further studies are needed to identify and characterize critical determinants in the M2-TMD to clarify how TMD composition and local lipid environments cooperatively regulate viral assembly and budding. Such insights may ultimately contribute to the development of antiviral strategies targeting the interaction between M2 and cellular membranes.

## Data Availability

The data presented in this study are available on request from the corresponding author.

## Supplementary Materials

The following supporting information can be downloaded a: https://www.mdpi.com/article/10.3390/v18010134/s1. Table S1: Primers used for the generation of M2-TMD mutant gene; Figure S1: Construction of M plasmids. Figure S2. Expression of M2 and its TMD mutants on cell surfaces.
