# Supplementary material for: Transmembrane Domain Length of Influenza a Virus M2 Does Not Determine Its Non-Lipid Raft Localization
Source: Viruses. 2026 Jan 21;18(1):134. doi: 10.3390/v18010134 (PMC12846675; doi:10.3390/v18010134)
Supplement: Supplementary file 1 [file viruses-18-00134-s001.zip › Figure S1.pdf]

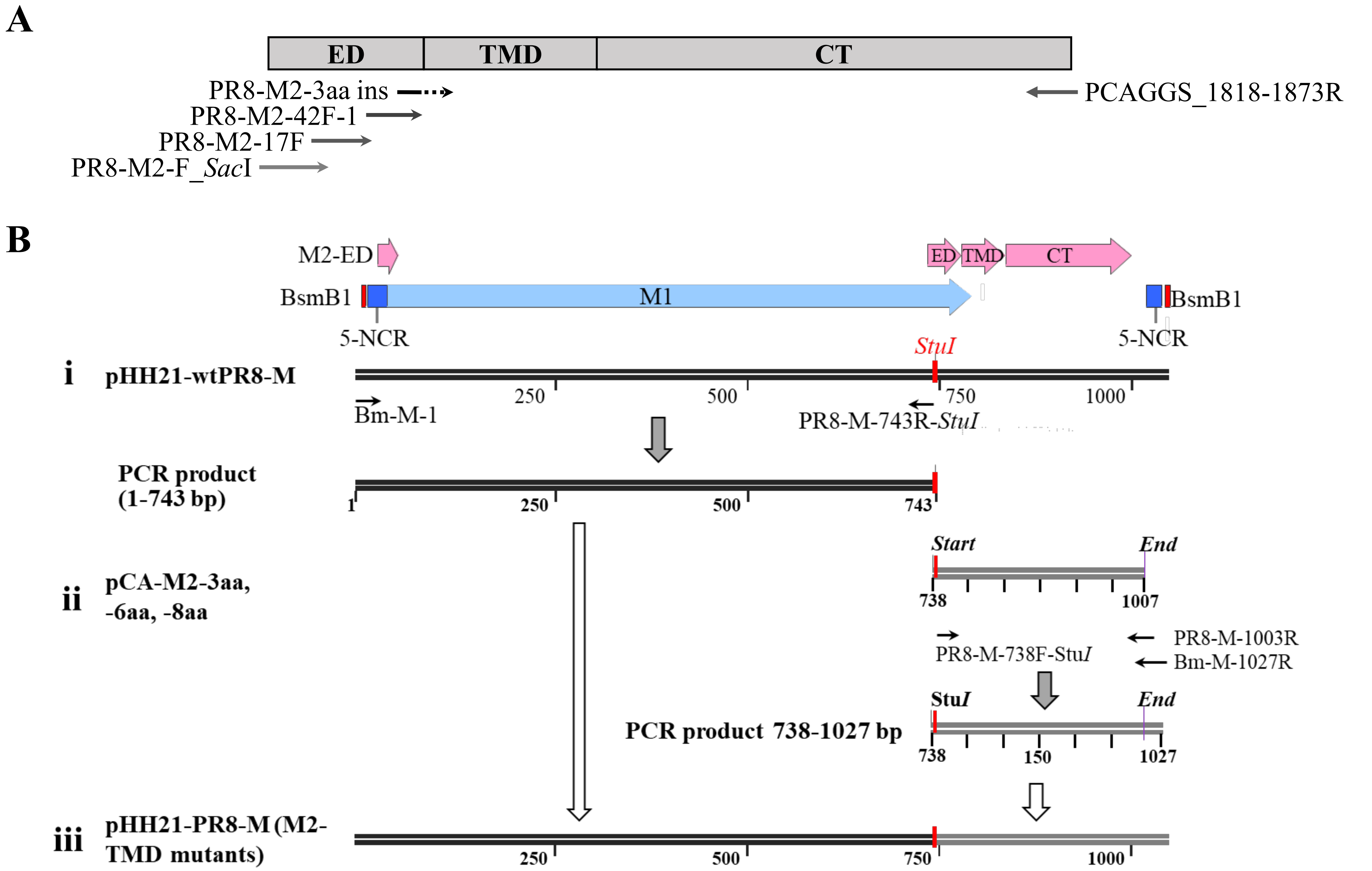

Figure S1. Construction of M plasmids. (A) series of overlapping primers shown in Table S1 were used to introduce mutations into the N-terminal region of M2-TMD using the pCA-wtM2 plasmid as a template. The figure depicts the construction of the pCA-M2-3aa plasmid. Other plasmids were also constructed in a similar way. **(B)** Schematic diagram showing the construction of pHH21 plasmids encoding M gene vRNA containing TMD mutations. (i) Schematic diagram showing the M gene encoding M1 (blue) and M2 (pink) proteins and restriction enzyme sites used to construct full length TMD mutant M genes. Bm-M-1 and PR8-M-743-*Stu*I primers were used to amplify a PCR fragment 1–743 bp. (ii) PCR fragment 738–1027 bp was amplified from expression plasmids pCA-M2-3aa, -6aa, and -8aa plasmids. (iii) The resultant fragments were digested with *BsmB1* and *StuI* and cloned into the pHH21 plasmid as described in detail in material and methods. Black bar indicates the M gene fragment (1–743 bp) derived from pHH21-PR8-M and gray bar indicates the fragment (738–1027bp) derived from pCA-M2-3aa, -6aa, or -8aa plasmids.
