# Supplementary material for: Transmembrane Domain Length of Influenza a Virus M2 Does Not Determine Its Non-Lipid Raft Localization
Source: Viruses. 2026 Jan 21;18(1):134. doi: 10.3390/v18010134 (PMC12846675; doi:10.3390/v18010134)
Supplement: Supplementary file 1 [file viruses-18-00134-s001.zip › Figure S2.pdf]

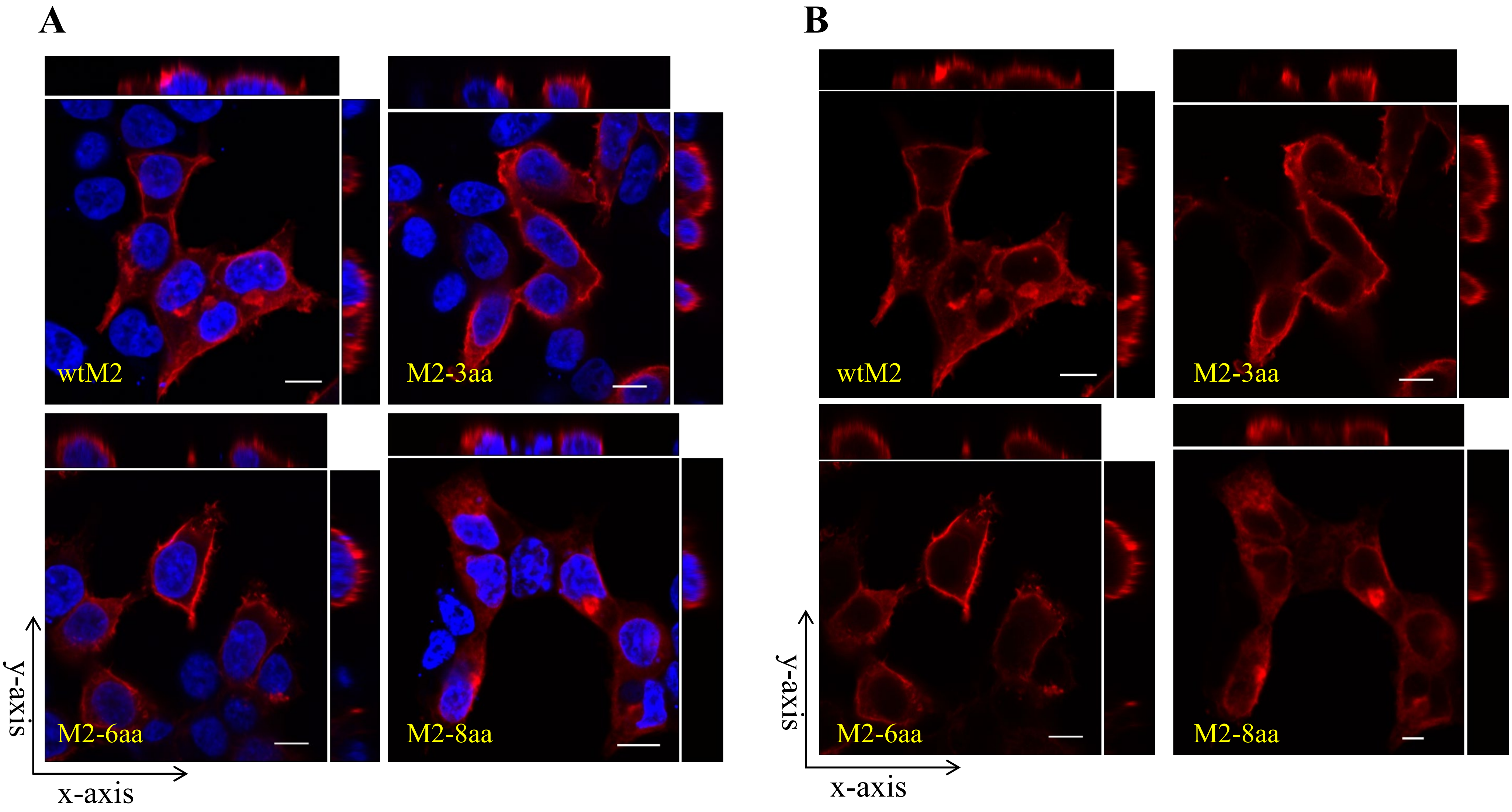

Figure S2. Expression of M2 and its TMD mutants on cell surfaces. Immunofluorescence staining of HEK293T cells transfected with expression plasmids described above. At 24 hrs post-transfection, cells were fixed, permeabilized, blocked, and stained with an anti-M2 monoclonal antibody and goat anti-mouse IgG (H+L) Alexa Fluor™ 594 secondary antibody. Nuclei were stained with DAPI. Images were acquired as z-stacks and representative slices showing the *xy, xz* and *yz* planes are presented. (**A**) merged image with DAPI (B) Image from "A" showing only M2 protein. Scale bars indicate 10 μm.
