# Supplementary material for: Transmembrane Domain Length of Influenza a Virus M2 Does Not Determine Its Non-Lipid Raft Localization
Source: Viruses. 2026 Jan 21;18(1):134. doi: 10.3390/v18010134 (PMC12846675; doi:10.3390/v18010134)
Supplement: Supplementary file 1 [file viruses-18-00134-s001.zip › Table S1.pdf]

Table S1. Primers used for the generation of M2-TMD mutant genes

Plasmid name	Primer Name	Template	Primer sequence (5`----->3`)
pCA-M2-3aa	PR8-M2_3aa ins	pCA-wtM2	ACGGAGCTCCCTCTC**GTCATTCTC**GCTATTGCCGC
	PR8-M2-42F-1		ATGGGGGTGCAGATGCAACGGTTCAAGTGATCCTCTC**GTCATTCTC**GC
	PR8-M2-17F		GGTCGAAACGCCTATCAGAAACGAATGGGGGTGCAGATGC
	PR8-M2-F_*Sac* I		ATTGAGCTCATGAGCCTTCTAACCGAGGTCGAAACGCC
	pCAGGS_1818-1837R		ATTAGCCAGAAGTCAGATGC
pCA-M2-6aa	PR8-M2_6aa ins	pCA-wtM2	ACGGAGCTCCCTCTC**GCTATTCTCGTCATTCTC**GCTATTGCCGC
	PR8-M2-42F-2		GGGGTGCAGATGCAACGGTTCAAGTGATCCTCTC**GCTATTCTCGTCATTCTC**GC
	PR8-M2-17F		GGTCGAAACGCCTATCAGAAACGAATGGGGGTGCAGATGC
	PR8-M2-F_*Sac* I		ATTGAGCTCATGAGCCTTCTAACCGAGGTCGAAACGCC
	pCAGGS_1818-1837R		ATTAGCCAGAAGTCAGATGC
pCA-M2-8aa	PR8-M2-42F-3	pCA-M2-3aa	GGGGTGCAGATGCAACGGTTCAAGTGATCCTCTC**ATTCTCGCTATTCTCGTCATTC**
	PR8-M2-17F		GGTCGAAACGCCTATCAGAAACGAATGGGGGTGCAGATGC
	PR8-M2-F_*Sac* I		ATTGAGCTCATGAGCCTTCTAACCGAGGTCGAAACGCC
	pCAGGS_1818-1837R		ATTAGCCAGAAGTCAGATGC
pHH-PR8M2 (M2-TMD mutants)	Bm-M-1	pHH21-PR8-M	TATTCGTCTCAGGGAGCAAAAGCAGGTAG
	PR8-M-743R-*Stu* I		AGCAGGCCTGCAAATTTTCAAGAAGATC
	PR8-M-738F-*Stu* I	pCA-M2-3aa, pCA-M2-6aa, pCA-M2-8aa	AATAGGCCTATCAGAAACGAATGG
	PR8-M-1003R		GAAACAAGGTAGTTTTTACTCCAGCTCTATGCTG
	Bm-M-1027R		ATATCGTCTCGTATTAGTAGAAACAAGGTAGTTTTT

The sequences in bold were used to introduce insertion mutations in M2-TMD, while the underlined sequences denote recognition sites for restriction endonucleases.
